# Low Complexity Robust Data Demodulation for GNSS

**DOI:** 10.3390/s21041341

**Published:** 2021-02-13

**Authors:** Lorenzo Ortega, Charly Poulliat, Marie Laure Boucheret, Marion Aubault Roudier, Hanaa Al-Bitar, Pau Closas

**Affiliations:** 1 Telecommunications for Space and Aeronautics Lab (TéSA), 31500 Toulouse, France; 2 INP-ENSEEIHT, University of Toulouse, 31000 Toulouse, France; charly.poulliat@enseeiht.fr (C.P.); marie-laure.boucheret@enseeiht.fr (M.L.B.); 3 CNES, 31400 Toulouse, France; Marion.Aubault@cnes.fr; 4 Thales Alenia Space, 31100 Toulouse, France; Hanaa.AlBitar@thalesaleniaspace.com; 5 Electrical and Computer Engineering Department, Northeastern University, Boston, MA 02115, USA; closas@ece.neu.edu

**Keywords:** GNSS, robust LLR, low complexity, SNR mismatch, noise estimation, bayesian inference, interference countermeasure

## Abstract

In this article, we provide closed-form approximations of log-likelihood ratio (LLR) values for direct sequence spread spectrum (DS-SS) systems over three particular scenarios, which are commonly found in the Global Navigation Satellite System (GNSS) environment. Those scenarios are the open sky with smooth variation of the signal-to-noise ratio (SNR), the additive Gaussian interference, and pulsed jamming. In most of the current communications systems, block-wise estimators are considered. However, for some applications such as GNSSs, symbol-wise estimators are available due to the low data rate. Usually, the noise variance is considered either perfectly known or available through symbol-wise estimators, leading to possible mismatched demodulation, which could induce errors in the decoding process. In this contribution, we first derive two closed-form expressions for LLRs in additive white Gaussian and Laplacian noise channels, under noise uncertainty, based on conjugate priors. Then, assuming those cases where the statistical knowledge about the estimation error is characterized by a noise variance following an inverse log-normal distribution, we derive the corresponding closed-form LLR approximations. The relevance of the proposed expressions is investigated in the context of the GPS L1C signal where the clock and ephemeris data (CED) are encoded with low-density parity-check (LDPC) codes. Then, the CED is iteratively decoded based on the belief propagation (BP) algorithm. Simulation results show significant frame error rate (FER) improvement compared to classical approaches not accounting for such uncertainty.

## 1. Introduction

Reliable position, navigation, and timing information is a demanded feature in new applications such as intelligent transportation systems (ITSs), automated aircraft landing, or autonomous unmanned ground/air vehicles. In such applications, the main source of positioning information is provided by Global Navigation Satellite Systems (GNSSs) [1,2,3], a technology that has attracted much interest in recent years and that is required to provide not only reliability and integrity, but also authentication of legitimate transmission [4]. The effects of interference, whether intentional or unintentional, can degrade GNSS receiver performance, sometimes to the point of causing denial of service or even counterfeit transmissions to control the receiver positioning solution. Several of these effects have been reported in the state-of-the-art [5,6,7,8,9,10,11,12]. Moreover, several interference countermeasures have been already proposed at different stages of the receiver including the antenna design [13,14,15,16,17,18], radio-frequency front-end [19,20], and signal processing [21,22,23,24,25,26,27,28,29,30,31]. However, a key part of GNSS receivers is the data demodulation stage, which allows recovering essential information. The latter has been long disregarded, with only a few articles [32,33,34,35] in the state-of-the-art, but may be a critical point under interference scenarios, being the principal object of this article.

In recent years, the organizations in charge of the design of the new generation of GNSS signals have decided to include modern error correcting codes (i.e., such as low-density parity-check (LDPC) or convolutional codes [36]) in order to enhance the data demodulation performance. In this context, the inputs to the corresponding soft decoding algorithms are the so-called log-likelihood ratio (LLR) values [37], which are classical sufficient statistics that are computed to feed the input of soft input channel decoders. Under the binary additive white Gaussian noise channel (BI-AWGN) assumption [36], the LLR associated with a transmitted coded bit can be shown to be a scaled version of the noisy observation of this coded bit, the scaling factor being proportional to the signal-to-noise ratio (SNR), or equivalently, inversely proportional to the channel noise variance. Usually, under the perfect channel state information (CSI) assumption [33], the noise variance is considered perfectly known, although in most real applications, noise variance has to be estimated. An estimation error induces an SNR mismatch, which in turn can lead to possible decoding performance loss. For instance, [38] investigated the performance of the belief propagation (BP) algorithm for decoding low-density parity-check (LDPC) codes over a BI-AWGN channel and with SNR mismatches. Based on this study, the design of irregular LDPC codes for BI-AWGN channels and SNR mismatches was later proposed in [39]. In both cases, the SNR mismatch was defined as an “SNR offset” η, referred to as the ratio of the real value of the variance $\sigma^{2}$ and the estimated value of variance ${\hat{\sigma }}^{2}$, which in turn was assumed to affect the entire codeword (i.e., block-wise estimation is considered).

However, there are systems such as GNSS for which, due to their low data rate, the variance $\sigma_{n}^{2}$ should be estimated symbol-per-symbol, and a precise model of the probability density function (pdf) of the received symbol can be derived. On the other hand, due to their low data rate, new simple methods to induce intentional interference and to nullify the decoding algorithm have been identified. As an example, we can mention pulsed jamming, which randomly affects some percentage of the codeword symbols [32]. The latter causes the symbol instantaneous variance $\sigma_{n}^{2}$ to be altered.

In [32], to compute the LLR under scenarios with interference, the variance was proposed to be symbol-wise estimated by the maximum likelihood (ML) principle. However, in general, this criterion is known to be computationally demanding and not accurate when a low number of samples per symbol are available. In this paper, focusing on GNSS systems, which are based on direct sequence spread spectrum (DS-SS) signals, we present the following contributions:

1. We derive a closed-form LLR expression under AWGN channels, which could be directly applied in the following cases: (i) The codeword data are demodulated over an open sky scenario with variations of the signal-to-noise ratio (SNR). (ii) The codeword data are demodulated over an interference scenario where an additive Gaussian noise is added to the entire codeword. To this end, we reformulate the problem of obtaining the LLR values by first computing the joint pdf of symbols and estimated variance, which is then marginalized in order to compute the desired LLRs used at the decoder. To implement such marginalization in practice, we propose to impose a conjugate prior distribution that allows for an analytic closed-form approximation that enables a reduced complexity implementation when compared to the ML solution. Then, assuming statistical knowledge of the estimation error of the noise power per symbol, a closed-from LLR approximation is derived. In our approach, we consider that the noise variance $\sigma^{2}$ is not perfectly known, but instead, symbol-wise independent estimations of this noise variance per symbol are available. We further assume a statistical distribution of such estimated variance. In this work, we model the variance of the *n*-th symbol $\sigma_{n}^{2}$ as a random variable, which is characterized by an inverse log-normal distribution (with the aim of taking advantage of the conjugate prior distribution) whose mean and variance are estimated at the decoder.
2. We derive a closed-form LLR expression that can be directly applied over a pulsed jamming scenario characterized by a small percentage of codeword symbols disrupted with extra Gaussian noise. Since the Gaussian distribution is known to not fit well the heavy tails caused by pulsed jamming [33], we propose to represent the received symbol distribution with a Laplacian distribution. Then, we compute the marginalized joint pdf of symbols and estimated variance in order to compute the desired LLRs used at the decoder. Again, we propose to compute the marginalization by imposing a conjugate prior distribution that allows for an analytic closed-form expression.

The paper is organized as follows: Section 2 presents the communication system models and the ML solution. Section 3 derives the closed-form expression based on conjugate priors when considering a system model with uncertain noise variance. In Section 4, we propose a closed-form LLR expression for pulsed jamming scenarios. A summary of the proposed methods and a comparison to state-of-the-art approaches are provided in Section 5. Then, in Section 6, results are presented and analyzed in the context of BP decoding of LDPC codes. Conclusions and perspectives are finally drawn in Section 7.

## 2. System Models and Assumptions

In this section, we present the system model for the three communication scenarios evaluated in this work. These are respectively the open sky scenario, Gaussian jamming, which disrupts the entire message, and pulsed jamming, which disrupts some symbols of the transmitted message. Moreover, in this section, we introduce the metric used for modern soft error correcting schemes such as turbo codes or LDPC codes. The soft inputs are the so-called log-likelihood ratio (LLR) values [37]. They are classical sufficient statistics used to feed the input of soft input channel decoders. Therefore, the LLR metric is not specific to the use of the LDPC codes or any other error correcting codes, but it is used as the mandatory input of any soft input decoding algorithm (e.g., LDCP codes, turbo codes, convolutional codes, etc.). Since in this work, we focus on the GNSS context, we considered LDPC codes as a benchmark due to their adoption by the GPS L1C signal. On the other hand, the obtained results are expected to be qualitatively the same for other kinds of error correcting codes using soft input decoding.

### 2.1. Open Sky Communications

Let us consider a DS-SS system. We represent the transmitted message as a binary vector $u={\left[u_{1},\ldots ,u_{K}\right]}^{⊤}$ of *K* bits. This message is encoded into a codeword $c={\left[c_{1},\ldots ,c_{N}\right]}^{⊤}$ of length N>K and mapped to binary-phase shift-keying (BPSK) symbols, referred to as $x_{n}=\mu \left(c_{n}\right)\in \left\{-1,1\right\},\forall n=1\cdots N$. *n* represents the symbol time index, and the mapping rule μ(.) is defined as $\mu \left(c_{n}\right)=1-2\;c_{n}$. Each symbol $x_{n}$ is then spread using a pseudo-random noise (PRN) sequence that can be expressed in vector form as $p_{n}\in R^{L}$, where *L* corresponds to the number of chips of the PRN sequence. Then, the transmitted symbol per coded bit is given by:

(1) $$x_{n}=x_{n}\cdot p_{n}\in R^{L},\;n=\left\{1,\ldots ,N\right\}\;,$$

where, by convention, vectors are defined as column vectors. Then, chip-level rectangular pulse shaping is used before transmission. The transmission channel is modeled as a time-varying binary-input AWGN noise channel. This class of channels is AWGN channels for which the noise process is a sequence of independent zero-mean Gaussian random variables with time-varying variance $\sigma_{n}^{2}=10^{-{\left(E_{s}/N_{0}\right)}_{n}/10}$, such that ${\left(E_{s}/N_{0}\right)}_{n}$ denotes the instantaneous SNR associated with the *n*-th transmitted symbol, and the signal amplitude is assumed to be normalized. Assuming perfect time and frequency synchronization, the received baseband symbol sequence at the chip-level can be written as:

(2) $$y_{n}=x_{n}+w_{n}\in R^{L},\;n=\left\{1,\ldots ,N\right\}\;,$$

where $w_{n}$∼$N(0,L^{2}\cdot \sigma_{n}^{2}I_{L})$, with $I_{L}$ being the identity matrix of size *L*. Thus, the noise power remains constant within the transmission of $x_{n}$, but it can change from one time instant *n* to another time instant $n^{\prime }\neq n$. Note that in most of the communications systems, the SNR is assumed to be constant along the entire codeword. However, since the GNSS system has a low date rate, small SNR variations can be appreciated between symbols.

#### LLR Expression

The LLR associated with the *n*-th symbol is defined as [36]:

(3) $$L_{n}=\ln\left(\frac{P(c_{n}=0|y_{n})}{P(c_{n}=1|y_{n})}\right)=\ln\left(\frac{P(x_{n}=1|y_{n})}{P(x_{n}=-1|y_{n})}\right)\;.$$

The obtained LLRs are then used to feed the input of the soft input channel decoder. When perfect CSI is assumed, the LLR can be trivially computed as:

(4) $$L_{n}=\ln\left(\frac{\frac{1}{\sqrt{2\pi \sigma_{n}^{2}}}e^{-\frac{{\left(y_{n}^{⊤}p_{n}-1\right)}^{2}}{2\sigma_{n}^{2}}}}{\frac{1}{\sqrt{2\pi \sigma_{n}^{2}}}e^{-\frac{{\left(y_{n}^{⊤}p_{n}+1\right)}^{2}}{2\sigma_{n}^{2}}}}\right)=\frac{2}{\sigma_{n}^{2}}y_{n}^{⊤}p_{n}\;,$$

which explicitly assumes that the noise variance is perfectly known at the receiver. In practice, this assumption does not hold, and $\sigma_{n}^{2}$ has to be estimated, typically relying on a point estimate ${\hat{\sigma }}_{n}^{2}$ per symbol. Thus, without statistical knowledge on the estimation error, the mismatched LLR is:

(5) $${\hat{L}}_{n}=\frac{2}{{\hat{\sigma }}_{n}^{2}}y_{n}^{⊤}p_{n}\;,$$

where ${\hat{\sigma }}_{n}^{2}$ is the noise variance estimate during the *n*-th symbol period. We underline that the simplest method to infer ${\hat{\sigma }}_{n}^{2}$ is to rely on the symbol point estimate of the SNR or $E_{s}/N_{0}$, since $\sigma_{n}^{2}=10^{-{\left(E_{s}/N_{0}\right)}_{n}/10}$. This symbol point estimator is usually computed from the narrowband-wideband power ratio (NWPR) algorithm [40]. Another possible method that has been shown to improve the data demodulation performance [32] when considering sufficiently large *L* could be obtained symbol-wise by applying the ML criterion. In that case, deriving the log-likelihood function and finding its roots [32] result in:

(6) $$L\left(x_{n},\sigma_{n}^{2}\right)≜-\ln\left(p\left(y_{n}|x_{n},\sigma_{n}^{2}\right)\right)=\frac{L}{2}\ln\left(2\pi \sigma_{n}^{2}\right)+\frac{∥y_{n}-x_{n}p_{n}{∥}^{2}}{2\sigma_{n}^{2}}$$

(7) $${\hat{\sigma }}_{n}^{2}=\frac{1}{L}{∥y_{n}-{\hat{x}}_{n}p_{n}∥}^{2}\;\mathrm{with}\;{\hat{x}}_{n}=\frac{y_{n}^{⊤}p_{n}}{L}\;.$$

However, ML estimates are known to provide efficient estimates asymptotically as L→∞. Consequently, when a small number of samples per symbol is available, these estimates and the resulting LLRs might not be accurate.

### 2.2. Communications under Gaussian Jamming

Under this particular scenario, a real-valued AWGN jamming that disrupts the entire codeword with a noise variance $\sigma_{I}^{2}$ is added to the previous scenario. Then, the received symbol sequence is:

(8) $$y_{n}=x_{n}+w_{n}+w_{I,n}\in R^{L},\;n=\left\{1,\ldots ,N\right\}\;,$$

where $w_{I,n}∼N\left(0,L^{2}\sigma_{I}^{2}I_{L}\right)$ is the statistical model of the jamming effect. Let us denote $w_{N_{0}+I,n}=w_{n}+w_{I,n}$ as the interference plus noise term, then $w_{N_{0}+I,n}∼N\left(0,L^{2}\left(\sigma_{n}^{2}+\sigma_{I,n}^{2}\right)I_{L}\right)$. Again, we assume that the interference plus noise power remains constant within the transmission symbol.

#### LLR Expression

The LLR associated with the *n*-th symbol when assuming perfect CSI is defined as:

(9) $$L_{n}=\frac{2}{\sigma_{n}^{2}+\sigma_{I}^{2}}y_{n}^{⊤}p_{n}=\frac{2}{\sigma_{{\left(N_{0}+I\right)}_{n}}^{2}}y_{n}^{⊤}p_{n}\;,$$

where the term $\sigma_{{\left(N_{0}+I\right)}_{n}}^{2}=\sigma_{n}^{2}+\sigma_{I}^{2}$ is assumed to be perfectly known. Note that this value is directly related to the instantaneous signal-to-noise plus interference ratio $\sigma_{{\left(N_{0}+I\right)}_{n}}^{2}=10^{-{\left(E_{s}/\left(N_{0}+I\right)\right)}_{n}/10}$, where the signal amplitudes are normalized. Considering a real scenario, the assumption of a perfectly known $\sigma_{{\left(N_{0}+I\right)}_{n}}^{2}$ does not hold, and we typically rely on a point estimator ${\hat{\sigma }}_{{\left(N_{0}+I\right)}_{n}}^{2}$ per symbol. Then, the mismatched LLR under Gaussian interference yields:

(10) $$L_{n}=\frac{2}{{\hat{\sigma }}_{{\left(N_{0}+I\right)}_{n}}^{2}}y_{n}^{⊤}p_{n}\;,$$

where the NWPR method or the ML method in (7) can be used as a point estimator of ${\hat{\sigma }}_{{\left(N_{0}+I\right)}_{n}}^{2}$. Note that if even (10) and (5) are conceptually different, the scenarios are practically the same since the main difference is the increased variance from $\hat{\sigma_{n}^{2}}$ to ${\hat{\sigma }}_{{\left(N_{0}+I\right)}_{n}}^{2}$. Then, the point estimator used to compute the LLRs has the same structure, i.e., the point estimator performs with similar precision independently of the scenario.

### 2.3. Communications under Pulsed Jamming

Under this scenario, some percentage *P* of transmitted symbol are disrupted by an extra AWGN with instantaneous noise variance $\sigma_{I}^{2}$ (i.e., *P* is the ratio between the number of symbols affected by the interference and the total number of symbols). Then, the received symbol sequence is modeled as:

(11) $$y_{n}=\left\{\begin{array}{cc} x_{n}+w_{n}\in R^{L}, & n\in Q\;,\\ x_{n}+w_{n}+w_{I,n}\in R^{L}\in R, & n\in S\;, \end{array}\right.$$

where $w_{I,n}$∼$N(0,L^{2}\sigma_{I}^{2}I_{L})$ is the statistical model of the jamming effect. Q is the set of bits not affected by the jamming noise, and S is the set of bits harmed with the jamming. Note that $\frac{\left|S\right|}{\left|Q|+|S\right|}=P$ is the duty cycle [41], where |·| denotes the cardinality number. Furthermore, we underline that the set of bits S is unknown, and it cannot be inferred frame-to-frame, i.e., we assume that the jamming pulsing patterns are pseudorandom.

#### LLR Expression

The LLR associated with the *n*-th symbol when assuming perfect CSI is defined as:

(12) $$L_{n}=\left\{\begin{array}{cc} \frac{2}{\sigma_{n}^{2}}y_{n}^{⊤}p_{n}\;, & n\in Q\;,\\ \frac{2}{\sigma_{{\left(N_{0}+I\right)}_{n}}^{2}}y_{n}^{⊤}p_{n}\;, & n\in S\;, \end{array}\right.$$

which explicitly involves the variance of the noise plus interference $\sigma_{{\left(N_{0}+I\right)}_{n}}^{2}$ being known. Moreover, this model assumes that the sets Q and S are also known at the receiver. In order to compute the LLR under more realistic scenarios, i.e., without perfect CSI, we can always use the NWPR method or the ML method as a point estimator of the variance. However, if we search for a low complexity method to estimate the variance, note that the lack of knowledge of the sets Q and S makes it difficult to find a simple model. One possible choice could be to approximate the system model in (11) by a simple equivalent Gaussian model:

(13) $$y_{n}=x_{n}+w_{G,n}\in R^{L},\;n=\left\{1,\ldots ,N\right\},$$

where $w_{G,n}∼N\left(0,L^{2}\sigma_{G,n}^{2}I_{L}\right)$. Note that this model seeks to better characterize the heavy tails within the observation model distribution due to the symbols disrupted by an extra Gaussian noise. Moreover, we underline that under this particular system model, we have an equivalent variance $\sigma_{G,n}^{2}=\left(1-P\right)\sigma_{n}^{2}+P\sigma_{I,n}^{2}$ yielding the LLR expression:

(14) $$L_{n}=\frac{2}{\sigma_{G,n}^{2}}\cdot y_{n}^{⊤}p_{n}\;.$$

On the other hand, we explicitly assume that $\sigma_{G,n}^{2}$ is known, which it is unlikely in real scenarios. One possible method to infer ${\hat{\sigma }}_{G,n}^{2}$ is to estimate the equivalent channel variance through the NWPR method. Furthermore, this model has been shown to not represent properly the heavy tails appearing in the distribution due to the codeword symbols disrupted by the the extra Gaussian interference. The latter can be solved by modeling the transmission channel with a distribution that better fits the heavy tails caused by the interference effects. A simple, yet practical, choice is to model this transmission channel as an additive real-valued Laplacian noise on the symbols. The intuition is that the Laplacian distribution models more accurately heavy-tailed effects than a Gaussian distribution does. Several works showed that jamming interferences generate data outliers, which can be statistically described by heavy-tailed distributions. Then, the symbol sequence can be modeled as:

(15) $$y_{n}=x_{n}+w_{L,n}\in R^{L},\;n=\left\{1,\ldots ,N\right\},$$

where $w_{L,n}$∼$L(0,c_{L,n})$ and $c_{L,n}=\frac{1}{\sqrt{2}}\cdot \sqrt{\left(1-P\right)\sigma_{n}^{2}+P\sigma_{I,n}^{2}}$. For this particular scenario, the LLR simplifies to:

(16) $$L_{n}=-\frac{|y_{n}^{⊤}p_{n}-1|}{c_{L,n}}+\frac{|y_{n}^{⊤}p_{n}+1|}{c_{L,n}}\;.$$

where the signal amplitude is assumed to be normalized. Note that we have assumed again that $c_{L,n}$ is known, which is unfortunately unrealistic.

From the above methods to estimate the LLR values, we can conclude that low complexity methods to infer the LLRs without assuming perfect CSI are missing in the GNSS literature. Therefore, the goal of the following sections is to propose new closed-form LLR expressions that can be used under theses particular scenarios and limited statistical CSI knowledge.

## 3. Closed-Form LLR Expression with Uncertain Noise Variance

In this section, we focus on the derivation of a closed-from LLR expression considering that the noise variance is not constant along the entire codeword; however, it can change from symbol-to-symbol, and it can be modeled as a random variable that follows a given statistical distribution. Note that, in order to derive an LLR expression that can perform properly under this transmission channel, the ML method was proposed in the previous section. Thus, in this section, we aim to propose an LLR expression that can reduce the complexity induced by the ML method. In Section 3.1, we derive a closed-form LLR expression considering statistical CSI, i.e., considering the knowledge of the noise variance distribution. Then, in Section 3.2, we show that this closed-form LLR expression can be used when no CSI is available at the receiver once the first and the second order moments of the SNR are estimated. Finally, we underline that this LLR expression can be directly applied when considering the communication system models described in Section 2.1 and Section 2.2, i.e., under the open sky scenario and the Gaussian jamming scenario.

### 3.1. Closed-Form LLR Expression with Statistical CSI on the Noise Variance

From a Bayesian perspective [42], since $\sigma_{n}^{2}$ and $\sigma_{{\left(N_{0}+I\right)}_{n}}^{2}$ are unknown quantities, they should be considered as random variables. Let us consider the case of $\sigma_{n}^{2}.$ All the statistically relevant information about $x_{n}$ and $\sigma_{n}^{2}$ is contained in their joint posterior distribution $p(x_{n},\sigma_{n}^{2}|y_{n})$. Assuming that $x_{n}$ and $\sigma_{n}^{2}$ are independent, we have:

(17) $$p\left(x_{n},\sigma_{n}^{2}|y_{n}\right)\propto p\left(y_{n}|x_{n},\sigma_{n}^{2}\right)p\left(x_{n}\right)p\left(\sigma_{n}^{2}\right)\;,$$

where the first term corresponds to the likelihood of the observations given unknowns and the second and third terms represent the a priori knowledge about $x_{n}$ and $\sigma_{n}^{2}$, respectively. Given (2), the likelihood probability distribution function (pdf) turns out to be a Gaussian distribution as follows:

(18) $$p\left(y_{n}|x_{n},\sigma_{n}^{2}\right)∼N\left(x_{n}\cdot p_{n},L^{2}\sigma_{n}^{2}I_{L}\right)\;.$$

Let us further denote the normalized output of the matched filter as $y_{n}=\frac{y_{n}^{⊤}p_{n}}{L}\in R$. We can then equivalently define the joint posterior pdf $p(x_{n},\sigma_{n}^{2}|y_{n})$ as:

(19) $$p\left(x_{n},\sigma_{n}^{2}|y_{n}\right)\propto p\left(y_{n}|x_{n},\sigma_{n}^{2}\right)p\left(x_{n}\right)p\left(\sigma_{n}^{2}\right)\;,$$

with:

(20) $$p\left(y_{n}|x_{n},\sigma_{n}^{2}\right)∼N\left(x_{n},\sigma_{n}^{2}\right)\;,$$

where the variance is unknown. According to the definition of the LLR in (3), we are interested in obtaining the marginal distribution of $x_{n}$:

(21) $$p\left(x_{n}|y_{n}\right)=\int_{0}^{\infty }p\left(x_{n},\sigma_{n}^{2}|y_{n}\right)\;d\sigma_{n}^{2}\;,$$

which substituted into (3) yields:

(22) $$L_{n}=\ln\left(\frac{\int_{0}^{\infty }p\left(x_{n}=1,\sigma_{n}^{2}|y_{n}\right)\;d\sigma_{n}^{2}}{\int_{0}^{\infty }p\left(x_{n}=-1,\sigma_{n}^{2}|y_{n}\right)\;d\sigma_{n}^{2}}\right)\;.$$

Assuming that the symbols are equiprobable, (22) can be further expanded, by applying (19), as:

(23) $$L_{n}=\ln\left(\frac{\int_{0}^{\infty }p\left(y_{n}|x_{n}=1,\sigma_{n}^{2}\right)p\left(\sigma_{n}^{2}\right)\;d\sigma_{n}^{2}}{\int_{0}^{\infty }p\left(y_{n}|x_{n}=-1,\sigma_{n}^{2}\right)p\left(\sigma_{n}^{2}\right)\;d\sigma_{n}^{2}}\right)\;.$$

A common approach in Bayesian analysis, when possible, is to select a prior distribution to be the conjugate of the likelihood distribution, which results in a closed-form expression for the a posteriori distribution that is of the same type as the a priori one [42]. To ease the closed-form derivation, we can apply a change of variable that would rather consider the precision $\lambda_{n}≜1/\sigma_{n}^{2}$ and its associated conjugate prior distribution than working directly with $p(\sigma_{n}^{2})$.

Leveraging known results in Bayesian analysis involving Gaussian distributions [42,43,44], the conjugate prior for $\lambda_{n}$ under a Gaussian likelihood model is given by the Gamma distribution:

(24) $$p\left(\lambda_{n}\right)=\Gamma \left(a_{n},b_{n}\right)≜\frac{1}{b_{n}^{a_{n}}\Gamma \left(a_{n}\right)}\lambda_{n}^{a_{n}-1}e^{-\lambda_{n}/b_{n}}\;,$$

where Γ(·) is the standard Gamma function with parameters $a_{n}$ and $b_{n}$. Note that $a_{n}$ and $b_{n}$ denote the shape parameter and the scale parameter of the Gamma distribution. Moreover, the products $a_{n}\cdot b_{n}$ and $a_{n}\cdot b_{n}^{2}$ represent the mean and the variance of the Gamma distribution, respectively. As a consequence of this choice for the prior distribution, the posterior distribution defined as in (19) becomes also a Gamma distribution whose parameters are updated from the prior to incorporate the knowledge from the observations, that is,

(25) $$p\left(x_{n},\lambda_{n}|y_{n}\right)=\frac{1}{\sqrt{2\pi }b_{n}^{a_{n}}\Gamma \left(a_{n}\right)}\lambda_{n}^{a_{n}-\frac{1}{2}}e^{-\lambda_{n}\left(\frac{1}{b_{n}}+\frac{{\left(y_{n}-x_{n}\right)}^{2}}{2}\right)}.\;$$

The marginal distribution of interest can be therefore obtained by solving the integral:

(26) $$p\left(x_{n}|y_{n}\right)=\frac{1}{\sqrt{2\pi }b_{n}^{a_{n}}\Gamma \left(a_{n}\right)}\int_{0}^{\infty }\lambda_{n}^{a_{n}-1/2}e^{-\lambda_{n}\left(\frac{1}{b_{n}}+\frac{{\left(y_{n}-x_{n}\right)}^{2}}{2}\right)}\;d\lambda_{n}\;,$$

which, as shown in Appendix A, yields the following parametric LLR expression:

(27) $$\begin{array}{c} L_{n}=-\left(a_{n}+\frac{1}{2}\right)\left[\ln\left(\frac{1}{b_{n}}+\frac{{\left(y_{n}-1\right)}^{2}}{2}\right)\right.\left.-\ln\left(\frac{1}{b_{n}}+\frac{{\left(y_{n}+1\right)}^{2}}{2}\right)\right]\;. \end{array}$$

This expression is therefore a function of the matched filter’s output and the parameters $\left(a_{n},b_{n}\right)$ characterizing $p(\lambda_{n})$ (or equivalently $p(\sigma_{n}^{2})$), which are assumed perfectly known if perfect statistical CSI regarding $\sigma_{n}^{2}$ is available (i.e., the knowledge of the complete pdf and its associated parameters). In most situations, such knowledge is not directly available at the receiver, but instead, a statistical characterization of the SNR can be assumed. Moreover, $p(\lambda_{n})$ is not usually characterized by a Gamma distribution, as discussed in the next section. Instead, the Gamma distribution is used here as a surrogate distribution for which associated hyperparameters are derived using moment matching to best fit the actual distribution. The following section discusses an approach to compute the LLR values in (27) under such statistical assumptions, showing how using a surrogate distribution can ease the derivation of a closed-form expression that is inexpensive to compute at the receiver.

### 3.2. Closed-Form LLR Approximation with First and Second Order Moments of the SNR

We assume here that the parameters defining $p(\lambda_{n})$ (or equivalently $p(\sigma_{n}^{2})$) in (24) are not available or perfectly known at the receiver, i.e., complete statistical CSI is not available. Instead, the distribution of an SNR estimate is available at the receiver. Note that the SNR is directly related to the energy per symbol-to-noise power spectral density ratio ($E_{s}/N_{0}$), which in this contribution is assumed to be modeled as a Gaussian random variable (see Appendix B) with the mean and standard deviation of the distribution defined as $\mu_{{\left(E_{s}/N_{0}\right)}_{n}}$ and $\sigma_{{\left(E_{s}/N_{0}\right)}_{n}}$. Note that the choice of the Gaussian distribution to model the random variable ${\left(E_{s}/N_{0}\right)}_{n}$ is imposed for the sake of tractability. In practice, that might not be the exact distribution, but in our experiments (reported in Section 6), we observed that the mean and the variance of the ${\left(E_{s}/N_{0}\right)}_{n}$ estimator appear to adequately characterize the distribution. As a consequence, we have that $\lambda_{n}=10^{{\left(E_{s}/N_{0}\right)}_{n}/10)}$ such that $p(\lambda_{n})$ follows a log-normal distribution, which can be approximated by a Gamma distribution in order to obtain a closed-form LLR expression through conjugate analysis, as explained earlier in this section. We propose here an entropy-minimization approach to approximate such a distribution with the Gamma distribution—thus benefiting from the conjugate prior analysis—of interest in (27). In this context, it will be shown that the assumption of perfect statistical CSI can be limited to some partial statistical CSI, i.e., the knowledge of first and second order moments of the random variable $\lambda_{n}$.

We minimize the Kullback–Leibler (KL) divergence [45] between the two distributions, to find the parameters *a* and *b* that better fit the original log-normal distribution (for the precision $\lambda_{n}$):

(28) $$\left({\hat{a}}_{n},{\hat{b}}_{n}\right)=\arg\min_{a,b}D_{KL}\left(\log N\left(\mu_{\lambda_{n}},\sigma_{\lambda_{n}}\right)\;\left|\right|\;\Gamma \left(a,b\right)\right)\;,$$

where $\mu_{\lambda_{n}}$ and $\sigma_{\lambda_{n}}$ represent the mean and the standard deviation of the log-normal distribution, respectively, with $\mu_{\lambda_{n}}=\frac{\mu_{{\left(E_{s}/N_{0}\right)}_{n}}\log_{e}\left(10\right)}{10}$ and $\sigma_{\lambda_{n}}=\frac{\sigma_{{\left(E_{s}/N_{0}\right)}_{n}}}{10}\log_{e}\left(10\right)$ (see Appendix B). Moreover, $a_{n}$ and $b_{n}$ represent the shape parameter and the scale parameter of the Gamma distribution, and $D_{KL}\left(\cdot ||\cdot \right)$ denotes the KL divergence. Following [46], the KL divergence in (28) can be computed as:

(29) $$\begin{array}{cc} \; & D_{KL}\left(\log N\left(\mu_{\lambda_{n}},\sigma_{\lambda_{n}}\right)\;\left|\right|\;\Gamma \left(a,b\right)\right)=\log\left(\Gamma \left(a\right)\right)\\ & +a\left(\log\left(b\right)-\mu_{\lambda_{n}}\right)-\frac{1}{b}\log\left(2\pi e\sigma_{\lambda_{n}}^{2}\right)+\frac{1}{b}\left(\mu_{\lambda_{n}}+\frac{\sigma_{\lambda_{n}}^{2}}{2}\right)\;, \end{array}$$

which, substituted in (28), can be used to show [46] that the values *a* and *b* minimizing the KL divergence can be approximated as:

(30) $${\hat{a}}_{n}\approx 1/\sigma_{\lambda_{n}}\;{\hat{b}}_{n}\approx \sigma_{\lambda_{n}}^{2}e^{\mu_{\lambda_{n}}+\frac{\sigma_{\lambda_{n}}^{2}}{2}}\;.$$

where we can note that the shape parameter ${\hat{a}}_{n}$ is related to the variance parameter of the log-normal distribution. Then, for a given ${\hat{a}}_{n}$, the scale parameter would adjust the mean parameter of the log-normal distribution. Recall that $a_{n}\cdot b_{n}$ and $a_{n}\cdot b_{n}^{2}$ give the mean and variance of the resulting Gamma distribution.

As a result, the closed-form LLR value under SNR estimation is given by:

(31) $$\begin{array}{c} {\hat{L}}_{n}=-\left({\hat{a}}_{n}+\frac{1}{2}\right)\left[\ln\left(\frac{1}{{\hat{b}}_{n}}+\frac{{\left(y_{n}-1\right)}^{2}}{2}\right)\right.\left.-\ln\left(\frac{1}{{\hat{b}}_{n}}+\frac{{\left(y_{n}+1\right)}^{2}}{2}\right)\right]\;. \end{array}$$

Two considerations are worth mentioning: (1) In contrast to (5), where a point estimate of the variance is used, (31) uses point estimates of the parameters of its distribution. Therefore, (31) accounts for the uncertainty in the estimated variance. (2) Different modeling choices for the distribution of the SNR estimates are possible (e.g., Gamma), in which case one could proceed similarly to find the parameters of the closest Gamma distribution by minimizing their KL divergence.

## 4. Closed-Form LLR Expression for Pulsed Jamming Scenarios

In this section, we focus on the derivation of a closed-form LLR expression that can be applied to the pulsed jamming scenarios. Under this particular transmission channel, some percentage of codeword symbols are corrupted by an extra Gaussian noise. In several scenarios, this extra noise can generate heavy tails in the observation sample distribution. Moreover, the corrupted symbols imply that the variance of the channel is not constant along the entire codeword. Then, an LLR expression that takes into account this uncertainty is required. Note that the ML method can be derived in order to compute an LLR expression that can perform properly under this transmission channel. However, in this section, we aim to provide an LLR expression that can reduce the complexity provided by the ML method. To this end, we consider the assumption made in (15) where a Laplacian distribution has been considered to describe the observation sample distribution. Then, in Section 4.1, we derive a closed-form LLR expression considering statistical CSI. Then, we show that this closed-form LLR expression can be used when no CSI is available at the receiver once the first and the second order moments of the SNR are estimated.

### 4.1. Closed-Form LLR Expression under the Likelihood Laplacian Assumption with Statistical CSI on the Noise Variance

Under this particular scenario, we propose to use a Laplacian distribution in order to model the normalized output of the matched filter $y_{n}=\frac{y_{n}^{⊤}p_{n}}{L}$. Thus, the likelihood pdf is given as follows:

(32) $$p\left(y_{n}|x_{n},c_{n}\right)∼L\left(x_{n},c_{n}\right)\;.$$

We can equivalently define the joint posterior pdf $p(x_{n},c_{n}|y_{n})$ as:

(33) $$p\left(x_{n},c_{n}|y_{n}\right)\propto p\left(y_{n}|x_{n},c_{n}\right)p\left(x_{n}\right)p\left(c_{n}\right)\;.$$

with $p\left(y_{n}|x_{n},c\right)∼L\left(x_{n},c_{n}\right)$ and $c_{n}$ is the shape parameter, which is assumed to be unknown. Following the same methodology as in Section 3 and considering that $x_{n}$ is equiprobable, the LLR can be computed as:

(34) $$L_{n}=\ln\left(\frac{\int_{0}^{\infty }p\left(y_{n}|x_{n}=1,c_{n}\right)p\left(c_{n}\right)\;dc_{n}}{\int_{0}^{\infty }p\left(y_{n}|x_{n}=-1,c_{n}\right)p\left(c_{n}\right)\;dc_{n}}\right)\;.$$

Now, we would like to identify a suitable conjugate prior distribution for p(c) in order to ease a closed-form derivation. First, we apply the following variable change $\rho_{n}≜1/c_{n}$. Thus, the likelihood distribution defined in Equation (32) can be re-parameterized as:

(35) $$L\left(\mu ,c_{n}\right)=\frac{1}{2c_{n}}e^{-\left(\frac{\left|y_{n}-\mu \right|}{c_{n}}\right)}=\frac{\rho_{n}}{2}e^{-\rho_{n}\left|y_{n}-\mu \right|}\;.$$

Leveraging known results in the Bayesian analysis [42,43,44], the conjugate prior for $\rho_{n}$ under a Laplacian likelihood model is given by the Gamma distribution:

(36) $$p\left(\rho_{n}\right)≜\frac{1}{b_{n}^{a_{n}}\Gamma \left(a_{n}\right)}{\rho_{n}}^{a_{n}-1}e^{-\rho_{n}/b_{n}}\;,$$

As a consequence of this choice of the prior, the posterior distribution becomes also a Gamma distribution whose parameters are updated from the prior to incorporate the knowledge from the observations:

(37) $$p\left(d_{n},\rho |y_{n}\right)≜\frac{1}{2b_{n}^{a_{n}}\Gamma \left(a_{n}\right)}\rho^{a_{n}}e^{-\rho (\frac{1}{b_{n}}+\left|y_{n}-\mu \right|)}\;.$$

The marginal distribution of interest can be therefore obtained by solving the integral:

(38) $$p\left(d_{n}|y_{n}\right)=\frac{1}{2b_{n}^{a_{n}}\Gamma \left(a_{n}\right)}\int_{0}^{\infty }\rho^{a_{n}}e^{-\rho (\frac{1}{b_{n}}+\left|y_{n}-\mu \right|)}d\rho ,$$

which, as shown in Appendix C, yields the following LLR closed-form expression:

(39) $$\begin{array}{c} L_{n}=-\left(a_{n}+1\right)\left[\ln\left(\frac{1}{b_{n}}+\left|y_{n}-1\right|\right)-\ln\left(\frac{1}{b_{n}}+\left|y_{n}+1\right|\right)\right]\;. \end{array}$$

where the parameters $\left(a_{n},b_{n}\right)$ characterizing $p(\rho_{n})$ (or equivalently, $p(c_{n}^{2})$) are assumed perfectly known. Note that the choice of $p(\rho_{n})$ as a Gamma distribution aims to ease the derivation of a closed-form LLR expression. However, we underline that $p(\rho_{n})$ does not necessarily follow a Gamma distribution in reality. In the following section, we propose an approach to compute the LLR values considering the previous assumptions.

### 4.2. Closed-Form LLR Approximation with First and Second Order Moments of the SNR

In this section, we assume that the parameters defining $p(\rho_{n})$ are not available at the receiver. Instead, we consider that the distribution of the SNR is available at the receiver. As in Section 3.2, we assume that the ${\left(E_{s}/N_{0}\right)}_{n}$ is a Gaussian random variable (see Appendix D) with the mean and standard deviation being $\mu_{{\left(E_{s}/N_{0}\right)}_{n}}$ and $\sigma_{{\left(E_{s}/N_{0}\right)}_{n}}$, respectively. Then, $\rho =\sqrt{2}\cdot 10^{{\left(E_{s}/N_{0}\right)}_{n})/20}$ follows a log-normal distribution. In order to benefit from the conjugate prior analysis, while leveraging a realistic model for the variance, a fitting from the log-normal distribution to a Gamma distribution is proposed in Section 3.2 through the KL divergence minimization (see Equation (29)). Similarly, in this case, we can approximate the values ${\hat{a}}_{n}$ and ${\hat{b}}_{n}$ with closed-form values given by:

(40) $$\hat{a_{n}}\approx 1/\sigma_{\rho_{n}}\;,\;\hat{b_{n}}\approx \sigma_{\rho_{n}}^{2}e^{\mu_{\rho_{n}}+\frac{\sigma_{\rho_{n}}^{2}}{2}}\;.$$

where the details to compute $\mu_{\rho_{n}}=\left(\mu_{{\left(E_{s}/N_{0}\right)}_{n}}\log_{e}\left(10\right)\right)/20$ and $\sigma_{\rho_{n}}=\left(\sigma_{{\left(E_{s}/N_{0}\right)}_{n}}/20\right)\log_{e}\left(10\right)$ are found in Appendix D. As a result, the closed-form LLR value is given by:

(41) $$\begin{array}{c} L_{n}=-\left({\hat{a}}_{n}+1\right)\left[\ln\left(\frac{1}{{\hat{b}}_{n}}+\left|y_{n}-1\right|\right)-\ln\left(\frac{1}{{\hat{b}}_{n}}+\left|y_{n}+1\right|\right)\right]\;. \end{array}$$

Note that thanks to the previous approximations, we can obtain a closed-form LLR expression that depends on the first and second order moments of the SNR, which appear in the closed forms in (40).

## 5. Summary of the State-of-the-Art and Proposed LLR Estimates

In this section, we aim to provide a summary of the state-of-the-art and proposed expressions for LLRs for each of the discussed scenarios. In Table 1, we report the LLR expressions analyzed in Section 2, Section 3 and Section 4. The LLR expressions are classified according to both the transmission scenario and the available channel state information knowledge. Moreover, all the proposed methods in this contribution are highlighted with blue color. Note that perfect CSI does not hold when real receivers are used. However, having LLR expressions in this context gives some theoretical insights when evaluating the performance. LLR expressions that can be implemented in real receivers are denoted with the label mismatched CSI and statistical CSI. Note that under those particular cases, the receiver requires to infer some parameters in order to compute the LLR values.

Note from Table 1 that under the open sky scenario, to compute the state-of-the-art LLR values, we need to have access to the symbol variance ${\hat{\sigma }}_{n}^{2}$. A first method to infer this variance is by using the narrowband-wideband power ratio (NWPR) method, which is used to infer the signal-to-noise ratio (SNR). Note that the SNR is usually inferred with a lower rate than the data symbols. Therefore, this method cannot infer properly ${\hat{\sigma }}_{n}^{2}$ under fast SNR variations. On the other hand, we underline that this method is required in the GNSS architecture, and therefore, no extra computation complexity is required. A second method to infer ${\hat{\sigma }}_{n}^{2}$ is the ML method. Note that this method can improve the accuracy of the LLR expression, but it also increases the complexity of the receiver since (7) needs to be solved for each symbol. Note that the previous methods are also the current solutions to infer the variance under the Gaussian jamming and the pulsed jamming scenarios, i.e., ${\hat{\sigma }}_{{\left(N_{0}+I\right)}_{n}}^{2}$ and ${\hat{\sigma }}_{G,n}^{2}$.

In this contribution, we aim to provide new LLR expressions that allow improving the performance without adding much computational complexity. Under the Gaussian channels, i.e., the open sky and Gaussian jamming scenarios, we propose a closed-form LLR expression (31), which allows improving the accuracy of the LLR expression since it takes into account the variations of the signal-to-noise ratio. Moreover, the extra computation complexity of this method only implies computing for each codeword (e.g., GPS L1C Subframe 2 is 12 s) the first and second order moments of the SNR. Note that the first moment is essentially the SNR estimation, which is required to compute the position velocity and time (PVT) solution, then the extra computational complexity is due to the computation of the second order moment.

Furthermore, we underline that (31) can perform properly under some Pulsed Jamming scenarios, as is shown in the following section. In particular, when *P* tends to one, the observation sample distribution converges to a Gaussian distribution, and (31) can accurately compute the LLR values.

Finally, the proposed closed-form LLR expression (41) allows improving the accuracy of the LLR expression under some specific pulsed jamming scenarios since it takes into account the fact that some symbols of the codeword are corrupted by an extra Gaussian noise. Note that this LLR expression involves an additional computational complexity, which is the computation of the second order moment of the SNR.

## 6. Results

In this section, we evaluate by simulation the achievable performance when using derived LLR expressions as given in the preceding section. In particular, as an example, we provide the frame error rate (FER) performance for GPS L1C Subframe 2 [47] (N=1200), which is based on an irregular LDPC code of rate 1/2 and decoded by the sum-product algorithm [48].

**Figure 1:**
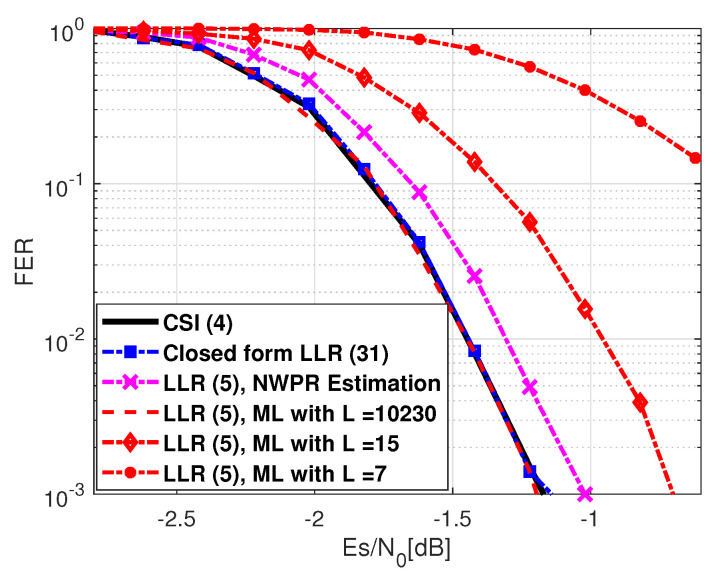
GPS L1C Subframe 2, FER considering a constant $\sigma_{n}^{2}$ for the entire codeword.

Several scenarios were considered. First, we considered an open sky scenario where the model of the variance $\sigma_{n}^{2}$ is constant for the entire transmission codeword. Moreover, a symbol-wise estimator based on the narrowband-wideband power ratio (NWPR) method [40] was used to estimate the ${\left(E_{s}/N_{0}\right)}_{n}$. Figure 1 shows the FER corresponding to the use of: (black) the perfect CSI-based LLR expression given by (4); (red) the LLR expression from (5) where ${\hat{\sigma }}_{n}^{2}$ is computed with the ML method and considering several *L* samples; (blue) the closed-form LLR approximation given in (31), considering that $\mu_{{\left(E_{s}/N_{0}\right)}_{n}}$ and $\sigma_{{\left(E_{s}/N_{0}\right)}_{n}}$ are estimates from the symbol-wise $E_{s}/N_{0}$ estimated values; (magenta) the LLR expression from (5) considering that ${\hat{\sigma }}_{n}^{2}$ is instantaneously computed from the $({E_{s}/N_{0})}_{n}$ estimates (provided by NWPR method) by applying (A4). Note that this last method is used by most of current commercial receivers, and it can be referred to as the state-of-the-art LLR expression.

We can see from Figure 1 that the performance when using the proposed LLR expression converges to that of the CSI based LLR solution. On the other hand, the LLR expression (5) where ${\hat{\sigma }}_{n}^{2}$ is directly estimated from the NWPR method finds an SNR mismatch, which leads to decoding performance losses. Considering the LLR expression based on the ML method for a large *L* (i.e., L= 10,230), ${\hat{\sigma }}_{n}^{2}$ is accurately estimated at the expense of an increase of the overall complexity. The proposed LLR expression converges to the perfect CSI-based LLR solution case. When a low number of samples per symbol are available, e.g., L=15 or L=7, then ${\hat{\sigma }}_{n}^{2}$ is poorly estimated, and the SNR mismatch can lead to large decoding performance loss. Moreover, in order to facilitate the comparison of the different LLR expressions, in Table 2, we provide the required $E_{s}/N_{0}$ to obtain a frame error rate (FER) of $10^{-2}$.

**Figure 2:**
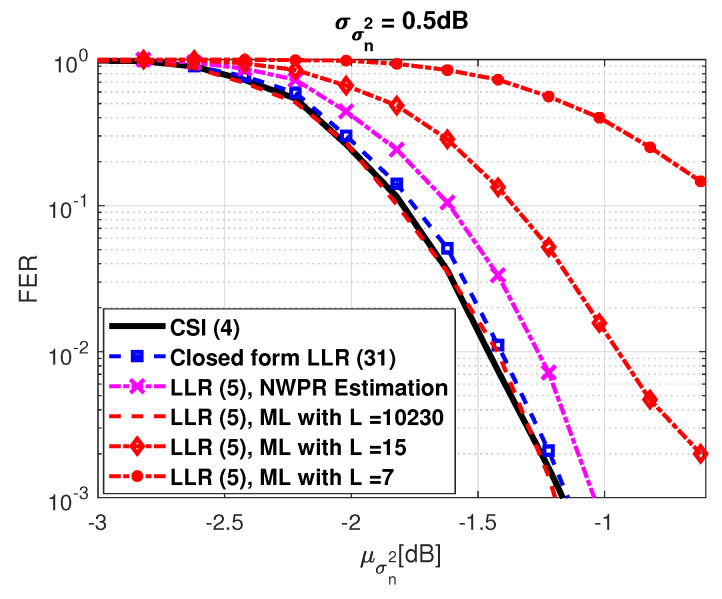
GPS L1C Subframe 2, FER considering a smooth variation of the variance $\sigma_{n}^{2}$ within the codeword.

A second experiment was done where smooth variations of the variance $\sigma_{n}^{2}$ were considered. The $\sigma_{n}^{2}$ variation can be characterized by a normal distribution whose mean and standard deviation are denoted as $\mu_{\sigma_{n}^{2}}$ and $\sigma_{\sigma_{n}^{2}}$, respectively. Again, the NWPR method was used to estimate ${\left(E_{s}/N_{0}\right)}_{n}$ symbol-wise. Notice from Figure 2 that the proposed LLR approximation converges to the perfect CSI-based LLR solution, whereas the ML method converges only when large *L* are considered, resulting in an increased complexity. Again, in order to facilitate the comparison between the different LLR expressions, we provide in Table 3 the required $E_{s}/N_{0}$ to obtain a frame error rate (FER) of $10^{-2}$.

**Figure 3:**
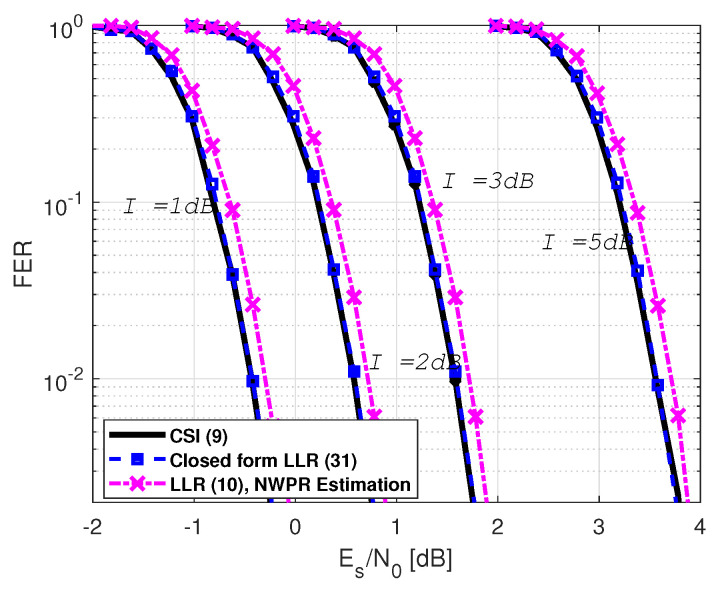
GPS L1C frame error rate under a Gaussian jamming.

In a third experiment, we assessed the performance considering a Gaussian jamming environment. The scenario sets a constant signal-to-noise plus interference ratio over the entire transmission codeword. We used the NWPR method to infer ${\left(E_{s}/\left(N_{0}+I\right)\right)}_{n}$ symbol-wise. Figure 3 shows the FER considering different interference powers (I=1 dB, I=2 dB, I=3 dB, and I=5 dB) corresponding to: the perfect CSI-based LLR values given by (9); the closed-form LLR approximation in (31), considering that $\mu_{{\left(E_{s}/\left(N_{0}+I\right)\right)}_{n}}$ and $\sigma_{{\left(E_{s}/\left(N_{0}+I\right)\right)}_{n}}$ are estimates from the symbol-wise ${\left(E_{s}/\left(N_{0}+I\right)\right)}_{n}$ estimates values; the LLR solution from (10) considering that ${\hat{\sigma }}_{{\left(T+I\right)}_{n}}^{2}$ is instantaneously computed from the ${\left(E_{s}/\left(N_{0}+I\right)\right)}_{n}$ estimates provided by NWPR method. We can see that the proposed closed-form LLR expression performance converges to the CSI LLR solution independently of the interference power. This solution improves the FER provided by just considering the NWPR method. On the other hand, even if the ML curves are not included in this figure (for the sake of the clarity of the plots), the same results as the previous experiments were found. The required $E_{s}/N_{0}$ to obtain an FER of $10^{-2}$ is included in Table 4.

**Figure 4:**
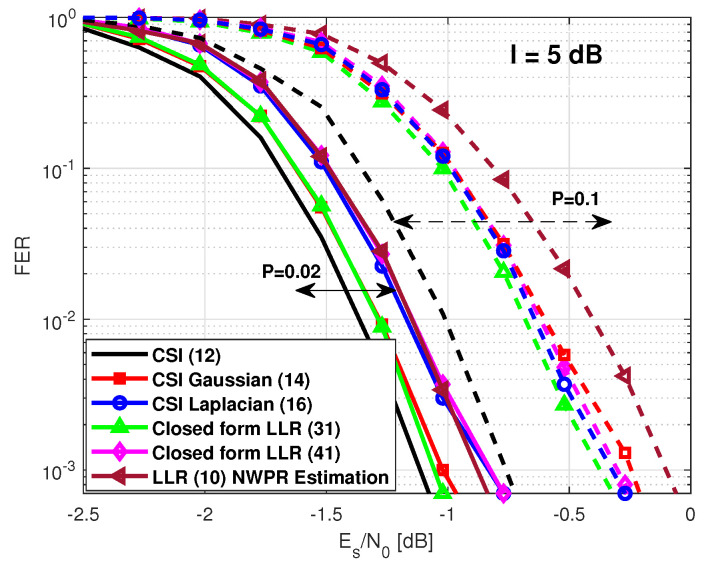
GPS L1C frame error rate under a pulsed jamming with *I* = 5 dB.

Finally, we assess the performance considering a pulsed jamming environment. Several scenarios were considered. First, we focus on scenarios with low values of *P*. Note that with intermediate values of *P*, it is simpler to detect the jammer, and other counter-measurements in the previous stage of the receiver chain can be applied. We consider a scenario where a jammer device disrupts with an extra Gaussian noise of 5 dB to each symbol ∈S. Again, a symbol-wise estimator based on the (NWPR) method was used to estimate the ${\left(E_{s}/\left(N_{0}+I\right)\right)}_{n}$. Moreover, we included the solutions of the LLR expressions (14) and (16) considering perfect knowledge of $\sigma_{G,n}^{2}$ and $C_{L,n}$. In Figure 4, the FER for the cases with P=0.02 and P=0.1 is illustrated. Since the power of the jamming is not powerful enough to generate large heavy tails in the observation sample distribution, the closed-form LLR expression based on the Laplacian distribution (41) performs worse than the closed-form LLR expression provided in (31). Moreover, we note that the closed-form expression (31) converges to the LLR solution (14) with perfect CSI and that the closed-form expression (41) converges to the LLR solution (16) with perfect CSI. The required $E_{s}/N_{0}$ to obtain an FER of $10^{-2}$ is included in Table 5.

**Figure 5:**
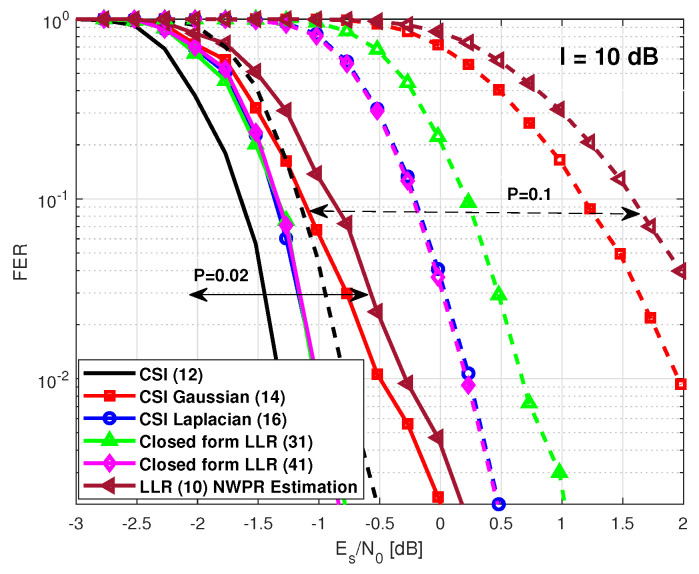
GPS L1C frame error rate under a pulsed jamming with I = 10 dB with low values of *P*.

In Figure 5, we illustrate the FER performance considering a scenario where the jammer device disrupts with an extra Gaussian noise of 10 dB. Note that the selected values are P=0.02 and P=0.1. In this particular case, the closed-form LLR expression based on the Laplacian distribution (41) performs better than the Gaussian approach. This is due to the fact that the jammer disrupts the codeword symbols with enough power to caused heavy tails in the observation sample distribution. Thus, the Laplacian distribution fits better to this particular problem. Moreover, we note that the closed-form expression (41) converges to the LLR solution (16) with perfect CSI. On the other hand, the closed-form expression (31) outperforms the LLR (14) with perfect CSI, showing the robustness of the solution.

**Figure 6:**
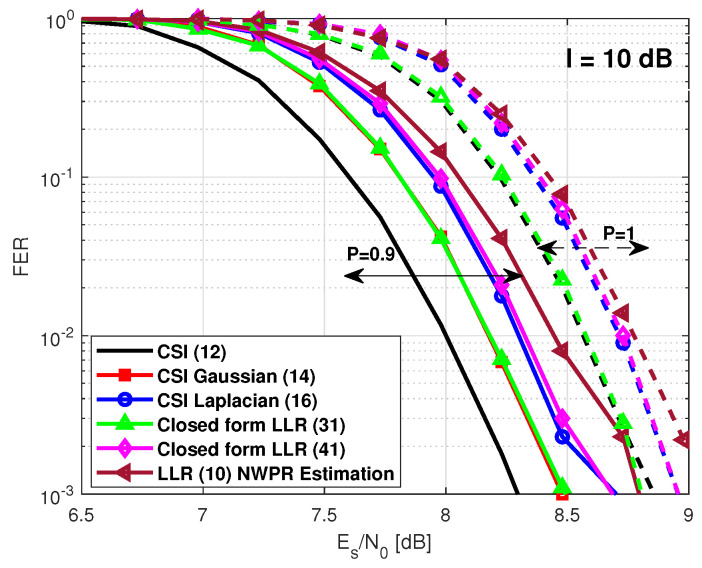
GPS L1C frame error rate under a pulsed jamming with I = 10 dB with high values of *P*.

We note that with values P∈(0.7–1), the observable sample distribution starts to converge to a Gaussian form. Then, the closed-form LLR expression based on the Gaussian approach (31) provides better FER performance than that obtained using a closed form LLR expression based on the Laplacian approach (41). The latter is illustrated in Figure 6 for P=0.9. In this figure, we also illustrate the case for P=1. Note that under this particular scenario, the observable sample distribution is Gaussian, and the closed-form LLR expression based on the Gaussian approach converges to the CSI-based LLR value solution. Furthermore, independently of the scenario, the proposed closed-form LLR expression performs better than the LLR solution from (10) considering that ${\hat{\sigma }}_{{\left(T+I\right)}_{n}}^{2}$ is instantaneously computed from the NWPR method. The required $E_{s}/N_{0}$ to obtain an FER of $10^{-2}$ is included in Table 6.

Finally, We underline that the proposed LLR closed-form expressions do not perform well with values P∈(0.2–0.7) since the Gaussian and the Laplacian model do not fit properly the observation sample distribution. Under these scenarios, it is recommended to use a more complex method, such as the ML, in order to infer the LLR values. On the other hand, we would like also to underline that with such a percentage of the values of the symbols disrupted by a jammer, it is simpler to detect the jammer and to include other signal processing countermeasures in order to clean the intentional interference.

## 7. Conclusions

In this paper, we address the issue of computing a closed-form LLR value under three particular GNSS scenarios: (i) open sky scenarios with smooth variations of the SNR, (ii) additive Gaussian noise interference, and (iii) pulsed jamming. Moreover, we assume a more realistic signal model, which considers SNR uncertainty. Since the GNSS system is a low data rate DS-SS system, it allows for symbol-wise estimation of the noise variance, thus providing information of the statistical distribution of the noise power. We reformulate the problem of computing the LLR values by modeling the variance as a random variable, which can be characterized by an inverse log-normal pdf whose mean and variance are considered known or well estimated. A Bayesian approach is taken in order to compute the joint posterior distribution of the variance and the transmitted symbol. This joint posterior is then marginalized to compute the LLR expression of interest. To compute the marginalized distributions, an analytic closed-form solution based on conjugate prior analysis is presented. The FER for the particular case of the irregular LDPC code of GPS L1C Subframe 2 is computed under the iterative BP decoding algorithm. The results show that the proposed LLR expression (31) enables reaching the performance of the CSI solution under the open sky scenario, improving the performance provided by the state-of-the-art solution (≈0.15–0.2 dB). This result is also verified when a Gaussian jamming disrupts the entire codeword transmission. Several examples of the FER considering a pulsed jamming scenario are also presented in order to compare the performance between the proposed LLR expressions and the current solution of the state-of-the-art. Results show that since these solutions fit more properly the transmission channel, they improve the FER with respect to the state-of-the-art solutions. Finally, this research shows that the proposed LLR expression enables improving the FER performance while involving a reasonable computational complexity increase.

## Figures and Tables

**Table 1:** Summary of the evaluated LLR methods (the contributions in this paper appear in blue). NWPR, narrowband-wideband power ratio.

	Scenarios		
Type of CSI Used	Open Sky Scenario	Gaussian Jamming Scenario	Pulsed Jamming Scenario
Perfect CSI	Equation (4)	Equation (9)	Equation (9) Approx (14) Approx. (16)
Mismatched CSI using NWPR estimates	Equation (5) with ${\hat{\sigma }}_{n}^{2}$ estimated with NWPR	Equation (10) with ${\hat{\sigma }}_{{\left(N_{0}+I\right)}_{n}}^{2}$ estimated with NWPR	Equation (10) with ${\hat{\sigma }}_{G,n}^{2}$ estimated with NWPR
Mismatched CSI using ML estimates	Equation (5) with ${\hat{\sigma }}_{n}^{2}$ estimated with ML	Equation (10) with ${\hat{\sigma }}_{{\left(N_{0}+I\right)}_{n}}^{2}$ estimated with ML	Not evaluated
Statistical CSI using proposed approx. and related parameter estimates	Closed-Form (41)	Closed-Form (31)	Closed-Form (31) Closed-Form (31)

**Table 2:** GPS L1C Subframe 2. $E_{s}/N_{0}$ to obtain a frame error rate (FER) of $10^{-2}$ considering a constant $\sigma_{n}^{2}$ for the entire codeword.

	CSI (4)	Closed-Form LLR (31)	LLR (5) with NWPR Estimation	LLR (5) ML and L = 10,230	LLR (5) ML and L = 15	LLR (5) ML and L = 7
$E_{s}/N_{0}$	−1.45 dB	−1.44 dB	−1.30 dB	−1.44 dB	−0.96 dB	0 dB

**Table 3:** GPS L1C Subframe 2. $E_{s}/N_{0}$ to obtain an FER of $10^{-2}$ considering a smooth variation of the variance $\sigma_{n}^{2}$ within the codeword.

	CSI (4)	Closed-Form LLR (31)	LLR (5) with NWPR Estimation	LLR (5) ML and L = 10,230	LLR (5) ML and L = 15	LLR (5) ML and L = 7
$E_{s}/N_{0}$	−1.45 dB	−1.44 dB	−1.26 dB	−1.44 dB	−0.94 dB	0.2 dB

**Table 4:** GPS L1C Subframe 2. $E_{s}/N_{0}$ to obtain an FER of $10^{-2}$ considering a Gaussian jamming, which harms the entire codeword.

	CSI (4)	Closed-Form LLR (31)	LLR (5) with NWPR Estimation
$E_{s}/N_{0}$ with I=1 dB	−0.44 dB	−0.43 dB	−0.20 dB
$E_{s}/N_{0}$ with I=2 dB	0.56 dB	0.57 dB	0.80 dB
$E_{s}/N_{0}$ with I=3 dB	1.56 dB	1.57 dB	1.80 dB
$E_{s}/N_{0}$ with I=5 dB	3.56 dB	3.57 dB	3.80 dB

**Table 5:** GPS L1C Subframe 2. $E_{s}/N_{0}$ to obtain an FER of $10^{-2}$ considering a pulsed jamming with I=5 dB.

	CSI (4)	CSI (14) Gaussian Approx.	CSI (16) Laplacian Approx.	Closed-Form LLR (31)	Closed-Form LLR (41)	LLR (5) NWPR Estimation
P=0.02	−1.36 dB	−1.28 dB	−1.17 dB	−1.28 dB	−1.15 dB	1.15 dB
P=0.1	−1 dB	−0.6 dB	−0.65 dB	−0.68 dB	−0.62 dB	−0.4 dB

**Table 6:** GPS L1C Subframe 2. $E_{s}/N_{0}$ to obtain an FER of $10^{-2}$ considering a pulsed jamming with I=10 dB.

	CSI (4)	CSI (14) Gaussian Approx.	CSI (16) Laplacian Approx.	Closed-Form LLR (31)	Closed-Form LLR (41)	LLR (5) NWPR Estimation
P=0.02	−1.32 dB	−0.5 dB	−1 dB	−1 dB	−1 dB	−0.3 dB
P=0.1	−0.8 dB	1.95 dB	0.23 dB	0.63 dB	−0.21 dB	2.5 dB
P=0.9	8 dB	8.17 dB	8.3 dB	8.17 dB	8.33 dB	8.45 dB
P=1	8.56 dB	8.56 dB	8.72 dB	8.57 dB	8.73 dB	8.77 dB

## Appendix A. Derivation of the LLR Expression with Variance Uncertainty under Conjugacy

This appendix derives a closed-form expression for the LLRs in (27), where a conjugate prior is considered. We aim to give a closed-form expression for the integral in (26).

Introducing the following change of variable,

(A1) $$z=\lambda_{n}\left[\frac{1}{b_{n}}+\frac{{\left(y_{n}-x_{n}\right)}^{2}}{2}\right]\to \frac{dz}{d\lambda_{n}}=\frac{1}{b_{n}}+\frac{{\left(y_{n}-x_{n}\right)}^{2}}{2}\;,$$

the integral in (26) is formulated as:

(A2) $$p\left(x_{n}|y_{n}\right)=\frac{{\left[\frac{1}{b_{n}}+\frac{{\left(y_{n}-x_{n}\right)}^{2}}{2}\right]}^{-(a_{n}+1/2)}}{\sqrt{2\pi }b_{n}^{a_{n}}\Gamma \left(a_{n}\right)}\int_{0}^{\infty }z^{\left(a_{n}-1/2\right)}e^{-z}\;dz\;.$$

Note that the integral term in (A2) is a constant term. Substituting (A2) in (3) yields:

(A3) $$\begin{array}{cc} L_{n} & =\ln\left(\frac{\frac{{\left[\frac{1}{b_{n}}+\frac{{\left(y_{n}-1\right)}^{2}}{2}\right]}^{-(a_{n}+1/2)}}{\sqrt{2\pi }b_{n}^{a_{n}}\Gamma \left(a_{n}\right)}}{\frac{{\left[\frac{1}{b_{n}}+\frac{{\left(y_{n}+1\right)}^{2}}{2}\right]}^{-(a_{n}+1/2)}}{\sqrt{2\pi }b_{n}^{a_{n}}\Gamma \left(a_{n}\right)}}\right)=-\left(a_{n}+\frac{1}{2}\right)\ln\left(\frac{\left[\frac{1}{b_{n}}+\frac{{\left(y_{n}-1\right)}^{2}}{2}\right]}{\left[\frac{1}{b_{n}}+\frac{{\left(y_{n}+1\right)}^{2}}{2}\right]}\right)\\ \; & =-\left(a_{n}+\frac{1}{2}\right)\left[\ln\left(\frac{1}{b_{n}}+\frac{{\left(y_{n}-1\right)}^{2}}{2}\right)-\ln\left(\frac{1}{b_{n}}+\frac{{\left(y_{n}+1\right)}^{2}}{2}\right)\right]\;, \end{array}$$

which gives the expression for the LLR as given in (27).

## Appendix B. Log-Normal Modeling of the Detail Estimate Considering a Gaussian Distribution

We assume that the ${\left(E_{s}/N_{0}\right)}_{n}$ estimate follows a Gaussian distribution. Let us denote the mean and the standard deviation of the distribution as $\mu_{{\left(E_{s}/N_{0}\right)}_{n}}$ and $\sigma_{{\left(E_{s}/N_{0}\right)}_{n}}$. Then, the variance $\sigma_{n}^{2}$ can be computed as a function of the energy per symbol $E_{s}$ and the noise density $N_{0}$, yielding:

(A4) $$\sigma_{n}^{2}=10^{-{\left(E_{s}/N_{0}\right)}_{n}/10}\Rightarrow \lambda_{n}=10^{{\left(E_{s}/N_{0}\right)}_{n}/10}\;.$$

Let us denote the auxiliary variable as $Y=({\left(E_{s}/N_{0}\right)}_{n})/10)$, then:

(A5) $$\lambda_{n}=10^{Y}∼\log_{10}N\left(\mu_{{\left(E_{s}/N_{0}\right)}_{n}}/10,\sigma_{E_{s}/N_{0}}^{2}/100\right)\;.$$

Since the log-normal distribution is defined with the logarithm base *e*, the following logarithm base change rule is applied:

(A6) $$\log_{10}\lambda_{n}=\frac{\log_{e}\left(\lambda_{n}\right)}{\log_{e}\left(10\right)}\to \log_{e}\left(\lambda_{n}\right)=\log_{e}\left(10\right)\log_{10}\lambda_{n}$$

obtaining $\lambda_{n}∼\log_{e}N\left(\mu_{\lambda_{n}},\sigma_{\lambda_{n}}\right)$, whose mean and standard deviation are,

(A7) $$\mu_{\lambda_{n}}=\frac{\mu_{{\left(E_{s}/N_{0}\right)}_{n}}\log_{e}\left(10\right)}{10},\;\sigma_{\lambda_{n}}=\frac{\sigma_{{\left(E_{s}/N_{0}\right)}_{n}}}{10}\log_{e}\left(10\right)\;.$$

## Appendix C. Calculation of the LLR Values under Gamma Conjugacy and the Laplacian Likelihood

This Appendix derives the closed-form expression of the LLRs in (39), where a conjugate prior is considered. The operations are focused on solving the following integral

(38) $$p\left(d_{n}|y_{n}\right)=\frac{1}{2b^{a}\Gamma \left(a\right)}\int_{0}^{\infty }\rho^{a}e^{-\rho (\frac{1}{b}+\left|y_{n}-\mu \right|)}d\rho \;,$$

Introducing the auxiliary change of variable $Z=\rho_{n}\left[\frac{1}{b_{n}}+\left|y_{n}-\mu \right|)\right]$ with:

(A8) $$\frac{dZ}{d\rho_{n}}=\left[\frac{1}{b_{n}}+\left|y_{n}-\mu \right|)\right]\;,$$

the integral in (38) can be formulated as:

(A9) $$p\left(d_{n}|y_{n}\right)=\frac{{\left[\frac{1}{b_{n}}+\left|y_{n}-\mu \right|\right]}^{-(a_{n}+1)}}{2b_{n}^{a_{n}}\Gamma \left(a_{n}\right)}\int_{0}^{\infty }Z^{a_{n}}e^{Z}\;dZ\;,$$

where it is noted that the integral is a constant term that is denoted by the scalar *A*. Then, Equation (A10) yields:

(A10) $$p\left(d_{n}|y_{n}\right)=A\frac{{\left[\frac{1}{b_{n}}+\left|y_{n}-\mu \right|\right]}^{-(a_{n}+1)}}{2b_{n}^{a_{n}}\Gamma \left(a_{n}\right)}\;.$$

Substituting (A11) in Equation (3) yields:

(A11) $$\begin{array}{ccc} L_{n} & = & \ln\left(\frac{A\frac{{\left[\frac{1}{b_{n}}+\left|y_{n}-1\right|\right]}^{-(a_{n}+1)}}{2b_{n}^{a_{n}}\Gamma \left(a_{n}\right)}}{A\frac{{\left[\frac{1}{b_{n}}+\left|y_{n}+1\right|\right]}^{-(a_{n}+1)}}{2b_{n}^{a_{n}}\Gamma \left(a_{n}\right)}}\right)=\ln\left(\frac{{\left[\frac{1}{b_{n}}+\left|y_{n}-1\right|\right]}^{-(a_{n}+1)}}{{\left[\frac{1}{b_{n}}+\left|y_{n}+1\right|\right]}^{-(a_{n}+1)}}\right) \end{array}$$

(A12) $$\begin{array}{ccc} & = & -\left(a_{n}+1\right)\left[\ln\left(\frac{1}{b_{n}}+\left|y_{n}-1\right|\right)-\ln\left(\frac{1}{b_{n}}+\left|y_{n}+1\right|\right)\right]\;, \end{array}$$

which defines the expression of the LLR values in Equation (39).

## Appendix D. Normal Modeling of the Detail Estimate Considering a Laplacian Distribution

Under the assumption that ${\left(E_{s}/N_{0}\right)}_{n}$ follows a Gaussian distribution, let us denote the mean and the standard deviation of the distribution as $\mu_{{\left(E_{s}/N_{0}\right)}_{n}}$$\sigma_{{\left(E_{s}/N_{0}\right)}_{n}}$, respectively. Considering the Laplacian distribution in Equation (35), we can compute *c* as a function of the energy per symbol $E_{s}$ and the noise density $\left(N_{0}\right)$,

(A13) $$c_{n}=\frac{1}{\sqrt{2}}10^{-{\left(E_{s}/N_{0}\right)}_{n}/20}\;.$$

Then, the detail ρ in the equation is defined as:

(A14) $$\rho_{n}=\sqrt{2}\cdot 10^{{\left(E_{s}/N_{0}\right)}_{n}/20}\;.$$

Let us denote the auxiliary variable as $Y={\left(E_{s}/N_{0}\right)}_{n}/20$, then:

(A15) $$Y∼N(\mu_{{\left(E_{s}/N_{0}\right)}_{n}}/20,\sigma_{{\left(E_{s}/N_{0}\right)}_{n}}^{2}/400)\;.$$

Considering Equation (A14),

(A16) $$\rho_{n}=10^{Y}∼LogN\left(\mu_{{\left(E_{s}/N_{0}\right)}_{n}}/20,\sigma_{{\left(E_{s}/N_{0}\right)}_{n}}^{2}/400\right)\;.$$

Since the log-normal distribution is defined with the logarithm base *e*, the following logarithm base change rule is applied:

(A17) $$\log_{10}\rho_{n}=Y=\frac{\log_{e}\left(\rho \right)}{\log_{e}\left(10\right)}\to \log_{e}\left(\rho_{n}\right)=\log_{e}\left(10\right)\log_{10}\rho_{n}\;,$$

obtaining $\rho ∼Log_{e}N\left(\mu_{\rho_{n}},\sigma_{\rho_{n}}\right)$ whose mean and standard deviation are:

(A18) $$\mu_{\rho_{n}}=\left(\mu_{{\left(E_{s}/N_{0}\right)}_{n}}\log_{e}\left(10\right)\right)/20\;,$$

(A19) $$\sigma_{\rho_{n}}=\left(\sigma_{{\left(E_{s}/N_{0}\right)}_{n}}/20\right)\log_{e}\left(10\right)\;.$$
